# Transparent and credible practices under the microscope: a response to comments on Ihle et al.

**DOI:** 10.1093/beheco/arx026

**Published:** 2017-04-15

**Authors:** Isabel S. Winney, Malika Ihle

**Affiliations:** a Laboratoire Evolution & Diversité Biologique, Bâtiment 4R1, Université de Toulouse Paul Sabatier, 118 Route de Narbonne, 31062 Toulouse Cedex 09, France and; b Department of Animal and Plant Sciences, Alfred Denny Building, University of Sheffield, Western Bank, Sheffield S10 2TN, UK

Improving research practices is a community project, and as such we are excited to have received many responses to our paper (Ihle et al. 2017). Overall, preregistration provoked the most reactions, ranging from enthusiasm to reluctance to adopt the measures, and we will dedicate most of our response to this topic. The remaining comments focused on replications. Hatchwell (2017) highlighted that they provide the raw data to study the underlying cause of variation. Almost in answer to this, Parker and Nakagawa (2017) proposed that journals append each replication to the original study, and that researchers launch groups of related replications or “replication batteries,” so that we can begin to understand the variation in our systems. Before addressing some common misunderstandings about preregistration, we need to stress that, as publically funded scientists, we are accountable for the work that we do. Facing drastic funding cuts and the erosion of public confidence, this is not the time to argue about whether unreliable, irreproducible, and nonreplicable methods are more fun or bring us personal success; it is the time to embrace more rigorous scientific practices.

Preregistration was the most contentious proposal, with Koenig (2017) arguing that it stifles creativity and is unnecessary due to low type 1 error rates, Cockburn (2017) voicing doubts over preregistering studies that may change over time, and Blumstein (2017) expressing concerns about being scooped. We reiterate that the more freedom individuals have to reshape analyses, the higher the type 1 error rate climbs (Forstmeier et al. 2016). Consequently, the number of false positives from exploratory analyses will be higher than suggested by Koenig (2017), and it can only be reduced by documenting a priori what tests will be performed. Preregistration involves good planning, taking the creative time that previously would have been distributed throughout a project and re-allocating it to the start. Ultimately, this allows researchers to prevent mistakes and streamlines both analysis and writing (see Forstmeier 2017), which we find satisfying and rewarding. Preregistrations are not the end of the creative process: they are merely the part that we can guarantee is relatively free of bias. Exploratory analyses *can still be published*, right alongside pre-registered analyses. They are an important tool for generating new hypotheses, but they should be distinguished because they are, by definition, influenced by viewing the data. Lastly, researchers do not need to worry about protecting their ideas: preregistrations can be embargoed for up to 4 years on the Open Science Framework or as long as desired on AsPredicted.org. Besides, opening up preregistration (or part of it) right at conception could promote collaboration, mirroring the approach taken in epidemiology (Cockburn 2017).

With preregistration and replication, we can at last reduce the likelihood and mitigate the impact of type 1 errors and get on with the business of understanding variation. Writing preregistrations and replication reports, as well as protocols and codes for data preparation and data analyses, will improve the quality of scientific work, while opening up this documentation will allow researchers to get credit for the work they actually do. We encourage everyone to embrace measures that we know increase scientific rigor (e.g., Kidwell et al. 2016), and ask the unwilling not to impede their spread while others discover their benefits.

Eventually, “we must choose between what is easy and what is right” (to quote Dumbledore in *Harry Potter and the Goblet of Fire* [Rowling 2000]). As long as our goal is to aim to get closer to the truth, we know that improving our practices is part of our fundamental remit as scientists. Now that multiple tools and protocols have been developed to make that process easier, all that is left is for you to use them.

## FUNDING

M.I. was funded by a grant from the Natural Environment Research Council (NERC, MO 005941) awarded to T. Burke. I.S.W. was funded by an ERC grant to B. Pujol (ANGI 681484).
